# Deep Brain Stimulation of Caudal Zona Incerta and Subthalamic Nucleus in Patients with Parkinson's Disease: Effects on Voice Intensity

**DOI:** 10.4061/2011/658956

**Published:** 2011-10-19

**Authors:** Sofie Lundgren, Thomas Saeys, Fredrik Karlsson, Katarina Olofsson, Patric Blomstedt, Jan Linder, Erik Nordh, Hamayun Zafar, Jan van Doorn

**Affiliations:** ^1^Division of Speech and Language Pathology, Department of Clinical Sciences, Umeå University, SE-90185 Umeå, Sweden; ^2^Division of Otorhinolaryngology, Department of Clinical Sciences, Umeå University, SE-90185 Umeå, Sweden; ^3^Division of Clinical Neuroscience, Department of Pharmacology and Clinical Neuroscience, Umeå University, SE-90185 Umeå, Sweden; ^4^Rehabilitation Research Chair, King Saud University, Riyadh 11433, Saudi Arabia; ^5^Division of Clinical Oral Physiology, Department of Odontology, Umeå University, SE-90185 Umeå, Sweden; ^6^Department of Rehabilitation Sciences, College of Applied Medical Sciences, King Saud University, Riyadh 11433, Saudi Arabia

## Abstract

Deep brain stimulation of the subthalamic nucleus (STN-DBS) in patients with Parkinson's disease (PD) affects speech inconsistently. Recently, stimulation of the caudal zona incerta (cZi-DBS) has shown superior motor outcomes for PD patients, but effects on speech have not been systematically investigated. The aim of this study was to compare the effects of cZi-DBS and STN-DBS on voice intensity in PD patients. Mean intensity during reading and intensity decay during rapid syllable repetition were measured for STN-DBS and cZi-DBS patients (eight patients per group), before- and 12 months after-surgery on- and off-stimulation. For mean intensity, there were small significant differences on- versus off-stimulation in each group: 74.2 (2.0) dB contra 72.1 (2.2) dB (*P* = .002) for STN-DBS, and 71.6 (4.1) dB contra 72.8 (3.4) dB (*P* = .03) for cZi-DBS, with significant interaction (*P* < .001). Intensity decay showed no significant changes. The subtle differences found for mean intensity suggest that STN-DBS and cZi-DBS may influence voice intensity differently.

## 1. Introduction

Deep brain stimulation (DBS) in the subthalamic nucleus (STN) is an established and effective treatment for motor symptoms associated with Parkinson's disease (PD). Effects of STN-DBS on speech motor function, however, are less well defined and have been reported either as variable [1] or as an adverse side effect of the stimulation [2]. Recently, the caudal zona incerta (cZi) has been suggested as an alternative target in PD treatment [3, 4]. The effects of cZi-DBS on speech have not yet been reported in the literature. We have, therefore, decided to evaluate its effects on speech in conjunction with an on-going study on surgical outcomes of cZi-DBS, and to compare these with the effects of STN-DBS.

Speech problems have been reported to occur in 70% [5] and 89% [6] of PD patients at some stage during the course of the disease, with higher prevalence as the disease advances in its severity and/or its time course [7]. The cardinal symptoms of speech dysfunction in PD (hypokinetic dysarthria) are weak voice, variable speech rate, short rushes of speech, imprecise consonants, breathy and harsh voice, and monotonous pitch. Of those symptoms, the perception of weak voice has been corroborated by acoustic measures of vocal intensity that demonstrate that PD patients have reduced intensity compared with healthy controls [8]. Reduced intensity is associated with the early stages of speech deterioration in PD and is often severely affected when profound speech problems exist [9]. Studies have shown that PD patients have lower mean voice intensity and limited voice intensity range during normal conversation (see [10] for a review) as well as faster intensity decay than controls during vowel phonation and reading [11] and rapid syllable repetition [12]. Problems with voice intensity in PD have been attributed to the disease's effects on the respiratory and phonatory systems, resulting in decreased respiratory drive and incomplete closure of the vocal folds during speech production [10, 13]. 

Voice intensity plays a central role in the treatment of speech problems associated with PD to the extent that “the current primary focus in speech therapy for individuals with hypokinetic dysarthria is vocal loudness” [10, page 66]. The foremost example is the Lee Silverman Voice treatment (LSVT) that has attained Class II level of evidence for outcomes [14]. Considering the prominence of voice intensity in speech deterioration and its treatment in PD, the present study has focused on how it is affected by DBS. 

A review of STN-DBS as treatment for PD [2] cites frequencies of 4–17% of cases with speech deterioration as an adverse side effect of stimulation. Studies that have systematically studied speech outcomes in patients who have undergone STN-DBS treatment for PD have found that changes are variable from patient to patient and dependent on which speech features have been measured [15–29]. It is evident from those reports that STN-DBS affects speech features much less than it does limb and nonaxial motor symptoms, and recent results have suggested that DBS differentially affects the speech subsystems [25]. Despite the lack of consistent findings regarding effects of STN-DBS on speech “the diffuse clinical impression is that speech may often worsen after DBS” [15, page 366]. 

Studies that have specifically reported voice intensity outcomes following STN-DBS [15, 17, 23–26] all use different speech tasks and different types of measures (acoustic or perceptual), with varying followup durations after surgery, making it difficult to generalize findings on the effects of STN-DBS. However, regardless of differences in experimental detail, none of these studies has reported any statistically significant decrease of voice intensity as a stimulation effect of STN-DBS. Some have reported no significant changes [15, 17, 23], while others have found increases in mean intensity [16, 24–26]. 

STN is currently the most frequent target of choice when surgical treatment is employed for motor symptoms in PD. However, a recent report [3] has suggested that stimulation of the caudal zona incerta (cZi) may result in even better limb motor outcomes. That study reported that cZi should be stimulated in preference to the rostral region (rZi) in order to avoid the speech deterioration that was observed in some patients for whom electrodes were located in the rZi region. 

However, apart from a pilot study on perceptual speech features associated with cZi-DBS [30], there are, to date, no published reports that have systematically studied its effects on speech. It is important to investigate whether stimulation of this alternate target can demonstrate not only improved limb motor outcomes compared with STN-DBS, but also whether it is able to avoid the detrimental speech effects that can be associated with STN-DBS. The aim of this study was to compare effects of cZi-DBS and STN-DBS on voice intensity 12 months after surgery in patients with PD, using two different measures: mean voice intensity during a reading task and voice intensity decline during a rapid syllable repetition task.

## 2. Method

### 2.1. Patients

Sixteen consecutive patients (12 males and 4 females, aged between 49 and 72 years) with idiopathic PD were included in this prospective non-randomised study. The first eight patients had undergone STN-DBS (six bilateral and two left-side unilateral) and the following eight cZi-DBS (all bilateral). Seven of the eight patients in each group also participated in an accompanying study on the effects of DBS on articulatory precision [31]. The patients had been selected on clinical grounds for their suitability for DBS surgery, and not on their speech status. The patients were operated on between 2005 and 2007 (STN group) and 2008 and 2009 (cZi group). The clinical selection criteria for the patients' suitability for surgery were the same for both groups. The surgical procedures for the respective targets have been previously described in detail [3, 32]. An overview of the patients is presented in Table 1. The study has been approved by the Regional Ethical Review Board in Umeå (Dnr: 08-093M: 2008-08-18) and all examinations have been conducted in accordance with local and national guidelines for good clinical practice.

### 2.2. Speech Recording Procedure

The speech recordings were coordinated with the patients' scheduled neurological examinations and were conducted on the morning of the second day of an assessment protocol that extended over two consecutive days. In the preoperative testing condition (pre-op) the patients had undergone a levodopa challenge and were medicated with 1.5 times their normal morning dose. Testing was performed one hour after medication was taken in a defined on-state. The postoperative testing was conducted at the time of the one year followup after surgery (median 54 weeks after surgery, range 46–57 weeks) and was performed in two conditions: one with stimulator off for one hour before recording (off-stimulation) and one with stimulator on for one hour before recording (on-stimulation). The postoperative recordings were made in the morning when the patients were medicated with their usual postoperative dosage, which was less than preoperative levels for all patients. 

The recordings were made in a sound-treated booth, using a head-mounted microphone (Sennheiser MKE 2 P-C) that was calibrated using a purpose built calibration system [33], with a 15 cm mouth to microphone distance. The samples were recorded at 48 kHz sampling rate using a digital audio flash recorder (Marantz PMD 660) or in the case of some early recordings a digital audio tape recorder (Panasonic SV 3800). A calibration tone (80 dB, 1 kHz) was used at the beginning of each recording. 

Speech tasks selected from the recording protocol for this study consisted of a standard 90 word Swedish reading passage (The Appendix), and rapid syllable repetition of three syllables /pΛpΛpΛ*⋯*/, /tΛtΛtΛ*⋯*/ and /kΛkΛkΛ*⋯*/. In the early recordings, the patients were instructed to repeat each syllable sequence for as fast and for as long as they could. In the more recent recordings, the person conducting the test produced an auditory model of the task, and the patients first practised repeating the syllables evenly at normal tempo, before proceeding to their fastest possible tempo. Five STN patients received the earlier instructions in all testing conditions, while six cZi patients received the recent instructions in all conditions. The remaining three STN and two cZi patients received the earlier instructions for the preoperative test and the recent instructions for postoperative tests.

### 2.3. Intensity Measures

Two intensity measures were made from the recordings: average intensity during the reading passage and the regression slope of intensity decay during rapid syllable repetition. Intensity decay was calculated from the rapid syllable repetition rather than the reading passage because rapid syllable repetition has been found to be more consistent in differentiating PD patients from controls [12]. Average voice intensity (dB SPL) for the reading passage for each patient in each testing condition was extracted using the speech analysis software package Praat [34]. Intensity decay (dB SPL per second) was deduced from the linear regression slope of the intensity peaks in the vowels of consecutive syllables in the syllable repetition task. The experimenters who conducted the intensity measures (authors S. Lundgren and T. Saeys) were blinded to the target and stimulation conditions.

**Voice Intensity during Reading**

Each recording of the reading passage was edited manually to remove extraneous nonspeech sounds. Then an automatic Praat script was used to extract the intensity of every voiced frame of the passage. Mean intensity over the whole passage was then deduced from the individual frame measures.

**Intensity Decay during Syllable Repetition**

All syllable sequences were examined to determine their suitability for inclusion. The criterion for inclusion of a sequence was that it must have been produced within one breath, and that it must have consisted of at least eight measurable syllables, where a syllable was defined as measurable if it consisted of an increase of energy followed by a period of silence or reduced energy in the waveform [35]. Of a possible 144 sequences, 134 met the inclusion criteria. First and last syllables in each eligible sequence were then excluded to avoid start and end effects. Linear regression of the maximum intensity of each included syllable in the sequence against the time point for that intensity peak was performed in order to calculate the slope of the intensity decay. Finally, the intensity decay slopes of the three syllable repetition tasks were averaged for each patient in each testing condition.

The values reported are the group means of the intensity decay slopes for each testing condition, based on sequences of eight or more repeated syllables. Ten of a possible 144 sequences were excluded because they were too short (eight for one patient and two for another).

### 2.4. Reliability

For the reading passage, 50% of the samples were measured by the two experimenters. All the computed means were tested for interjudge reliability using Pearson product-moment correlation statistics (Pearson's *r*), and the reliability was significant (*r* = 0.83–1.00, *P* < .001). All of the syllable repetition sequences were measured separately by the same two experimenters. The measured syllable's time point pairs for each sequence were manually compared to verify that the correct syllables had been measured, and that the syllables corresponded to the syllable definition. Reliability tests using Pearson's *r* on the syllable intensities for 10% of the sequences showed that reliability was significant (*r* = 0.99, *P* < .001).

### 2.5. Statistical Analysis

Between- within analyses of variance (ANOVA) and post hoc *t*-testing were used to test for on-off stimulation as well as long-term stimulation effects of cZi-DBS and STN-DBS.

## 3. Results

### 3.1. Voice Intensity during Reading

**Figure 1:**
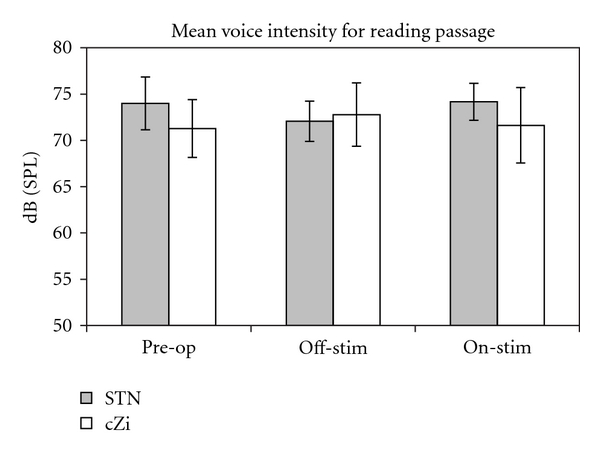
Voice intensity during reading for the STN and cZi groups, respectively, showing means and standard deviations for preoperative and postoperative off- and on-stimulation testing conditions.

Figure 1 shows the group means and standard deviations for voice intensity during reading for the STN and the cZi groups in each of the three testing conditions. Repeated measures ANOVA showed that there was a significant effect for testing condition for the STN group (*F*  (2,14) = 5.135, *P* = .02) but not for the cZi group (*F*  (2,14) = 2.147, *P* = .154).

**Off- versus on-Stimulation**

**Figure 2:**
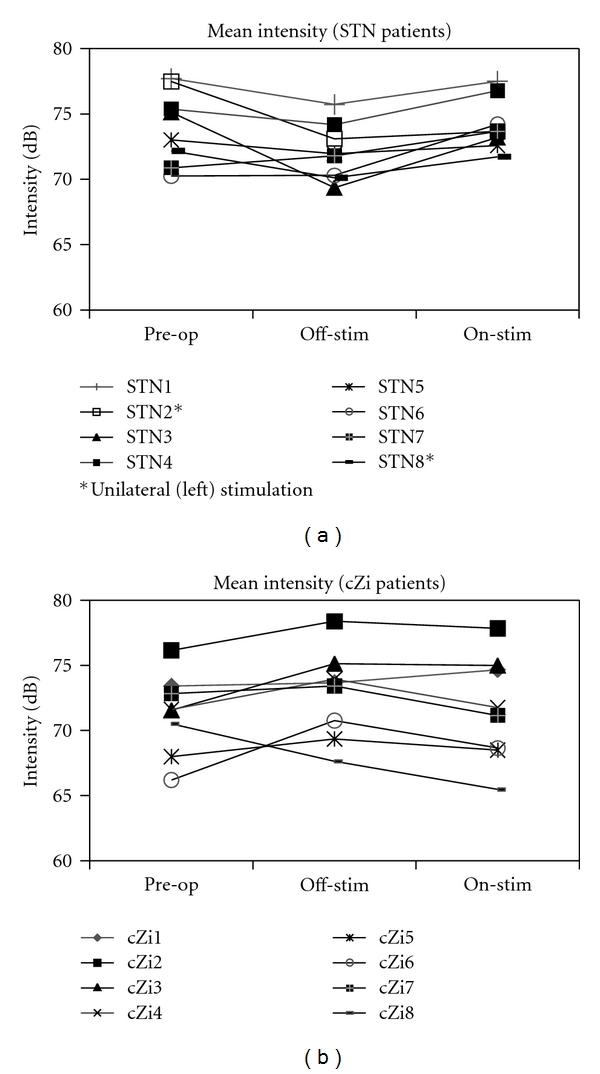
Mean intensity during reading for individual patients in STN and cZi groups.

It can be seen from Figure 1 that the effects of stimulation on mean intensity during reading are in different directions for the two groups, with an increase in intensity on-stimulation in the STN group and a decrease on-stimulation for the cZi group. Additionally, at an individual level, all eight STN-DBS patients showed the same pattern between off- and on-stimulation testing conditions, as did seven of eight cZi-DBS patients (see Figure 2). Paired *t*-testing revealed that for the STN group the on-stimulation mean (74.2 ± 2.0 dB) was significantly larger than off-stimulation (72.1 ± 2.2 dB; *t*(7) = 4.638, *P* = .002), while for the cZi group the on-stimulation mean (71.6 ± 4.1 dB) was significantly less than off-stimulation (72.8 ± 3.4 dB; *t*(7) = 2.697, *P* = .03). A between-within ANOVA confirmed that the interaction effect between group and testing condition was significant (*F*(1,14) = 27.258, *P* < .001).

**Long-Term Stimulation Effects**

Differences between the preoperative and the two postoperative testing conditions were examined for each group. It can be seen from Figure 1 that in each group the preoperative and on-stimulation mean intensities were very similar, and paired *t*-tests confirmed that there were no statistically significant differences between those conditions for either group. For the STN group, the mean intensity in the off-stimulation condition was significantly less than in the preoperative condition (72.1 dB, 74.0 dB; *t*(7) = 2.485, *P* = .04). There was, however, no significant difference between the mean intensities for those two conditions for the cZi group (71.3 dB, 72.8 dB; *t*(7) = −1.857, *P* = .106).

### 3.2. Intensity Decay during Syllable Repetition

**Figure 3:**
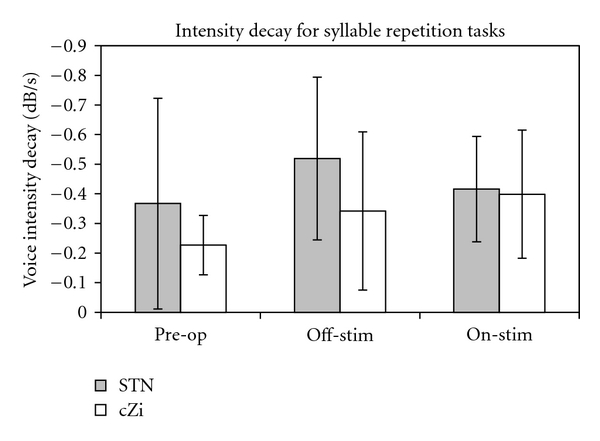
Syllable intensity decay (dB/s) for the STN and cZi groups respectively, showing means and standard deviations for preoperative and postoperative off- and on-stimulation testing conditions.

**Figure 4:**
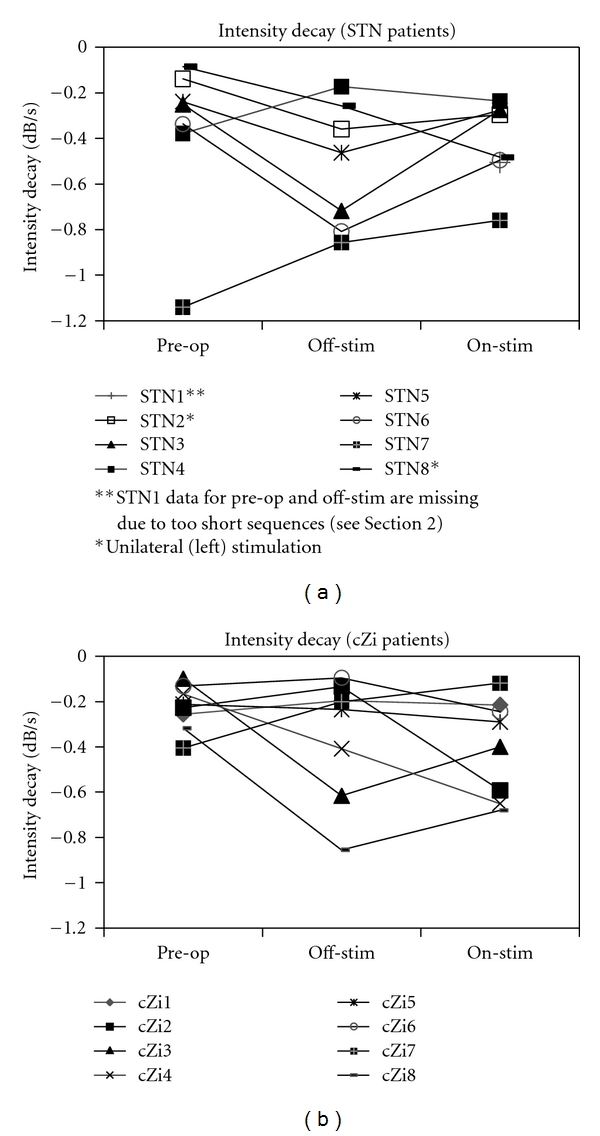
Intensity decay during syllable repetition for individual patients in STN and cZi groups.

Figure 3 shows the mean intensity decay slopes for each group in each condition. There was no overall significance of condition for either group, nor were there any significant differences between on- and off-stimulation testing conditions, or between preoperative and either postoperative condition. There was a high degree of individual variation within the groups that can be seen in Figure 4. For the STN group, four of the eight patients followed the trends for all three conditions shown in the group data while for in the cZi group only two of the eight patients had trends consistent with the group means. When comparing on- with off-stimulation testing conditions, five patients in the STN group had a shallower decline of voice intensity while for the cZi group five patients had a steeper decline. 

## 4. Discussion

This study is the first to report the effects of cZi-DBS on voice intensity in Parkinson's disease and compare them to effects of STN-DBS. Stimulation 12 months after surgery was investigated by comparing on- with off-stimulation conditions on the same day, while longer-term effects of surgery were studied by comparing the 12 months postoperative testing conditions with the preoperative condition. 

For on- versus off-stimulation 12 months after surgery, mean voice intensity during reading changed for both STN and cZi groups, but in different directions. The STN group showed an increase of 2.1 dB while the cZi group showed a reduction of 1.15 dB. Both results were statistically significant. The results at individual patient level were also notably consistent with the group result for all eight STN and 7 of 8 cZi patients. The results for intensity decay, on the other hand, were not significant for testing condition in either group and showed a high degree of individual variability for patients in both groups. The minor difference in the syllable repetition task instructions with a lack of a practice model mainly for the STN-DBS patients is unlikely to be the source of this variability since the results for the cZi group were equally or even more variable. 

The small statistically significant increase in mean intensity on-stimulation for the STN group in our study is consistent with one other report of a group of seven PD patients who had undergone STN-DBS. Dromey et al. [16] found a significant voice intensity increase of 1.1 dB in response to stimulation during a monologue speech task 6 months after surgery when the patients were in their medicated state. Like our study, the difference was also consistent at individual patient level. 

The difference in the response of voice intensity to stimulation between the two groups of patients in our study is unlikely to have been due to preoperative differences. At the time of surgery, the two groups did not differ significantly with regard to age, duration of disease since diagnosis, UPDRS III scores, or speech status according to UPDRS III Item 18 scores (Table 1) and preoperative dB (SPL) (Figure 1). It should, however, be noted that there were two patients with left-side unilateral stimulation in the STN group. Lateralisation effects of STN-DBS on various aspects of speech have been found [23, 26, 27], but the inspection of individual results for voice intensity in the current study (Figures 3 and 4) revealed no differences for the unilateral patients. Additionally, excluding the unilateral patients from the statistical analysis did not alter the statistical significance of any result.

It should be noted that the postoperative speech recordings in the current study were made when the patients were medicated at their usual postoperative dose levels. Since PD patients who have undergone DBS generally still take reduced doses of medication following surgery for best clinical outcome, testing with medication reflects their usual clinical status. Studies of DBS effects should preferably involve a medication-off state to capture the effects of stimulation alone as well as a medication-on state so that the usual clinical state is reflected in the experimental design. However, such a design necessitates testing patients when they are off-stimulation and without medication, a condition which involves considerable discomfort. Thus, we elected to test the patients only in the medicated conditions because we needed to consider carefully their limited availability for testing and their well being during that time. We would also have risked some patients being unable or declining to participate further in the study which could potentially have biased our longitudinal data collection. 

Studies that have reported results from all four testing conditions [15, 16, 22] may allow us to infer the effects of medication status when stimulation effects are being investigated. The findings are, however, not consistent. Smaller changes were found to be associated with stimulation when patients were medicated than when not medicated in two studies [15, 22]. Dromey et al. [16], on the other hand, found larger and more consistent changes with stimulation when patients were medicated. This inconsistency between studies is perhaps to be expected when one considers that studies of the effects of medication alone on speech in PD patients who are being treated pharmacologically are not conclusive, for example, [36–38]. The results of the current study have only been compared with studies that include equivalent testing conditions. 

While the differences in mean intensity between on- and off-stimulation conditions in both groups were found to be statistically significant, they were small, which is possibly because preoperatively there were only mild speech effects due to the disease. The magnitude of the differences for both groups is likely to be borderline for any clinical significance and it will be important to confirm this with a listening study in the future. While clinical significance is of utmost importance for the prognosis of the operation, subclinical differences may still have implications for understanding the effects of treatment on speech motor control.

Long-term effects of stimulation 12 months after surgery were studied by comparing preoperative and postoperative conditions. There was just one statistically significant result: a decrease in voice intensity during reading from the preoperative to the postoperative off-stimulation condition for STN-DBS, whereas for cZi-DBS there was a small but nonsignificant increase. The group means for voice intensity for postoperative on-stimulation testing conditions were very similar to the preoperative condition for both groups, with no statistically significant differences between those conditions. Measures of intensity decay were highly variable across patients and gave no statistically significant results. Our findings for long-term effects of STN-DBS on voice intensity contrast with those of Tripoliti et al. [25]. That study on a group of 32 STN-DBS patients reported a 7.4 dB increase in intensity during reading between preoperative and 12 months postoperative on-stimulation testing conditions when the patients were medicated.

It should be noted that the patients in our study had been subjected to a levodopa challenge for the preoperative test condition where their medication dosage was 1.5 times higher than usual. They were, however, all tested during their defined on-state. Thus the results of the long-term effects of stimulation need to be interpreted with some caution. However, if the decrease from preoperative to postoperative off-stimulation testing conditions for the STN group were to be explained by the fact that the preoperative medication dose was higher than the usual dosage, then the same finding should be expected for the cZi group, but this was not the case. However, before one could explain the difference as a long-term surgical effect that differs between the two surgical targets, larger group sizes will be required to confirm the finding. 

In summary, the most notable findings in our comparison of the effects of cZi-DBS with STN-DBS for PD patients were small on-off stimulation differences in different directions for mean voice intensity during reading 12 months after surgery. While the magnitudes of the differences were small, they were statistically significant and their consistency at individual patient level is noteworthy. Measures related to the underlying physiological responses could be used to determine if these changes are reflected in the respiratory system and vocal fold adduction, both of which can result in changes to vocal intensity. A recent study on the effects of STN-DBS on respiratory measures [13] has showed considerable interpatient variability for measures that reflect respiratory drive and degree of vocal fold adduction. While respiratory measures for patients in the current study were not recorded, it will be possible to further investigate their vocal fold adduction and intensity changes during sustained vowel phonation using recordings from transnasal laryngoscopic and electroglottographic recordings that were made as part of the larger recording battery.

## 5. Conclusion

This study investigated effects on voice intensity of cZi-DBS, a recently proposed alternative to STN-DBS as a surgical treatment of PD, and compared them with those found for STN-DBS. The results showed that the stimulation response of mean voice intensity during reading was different for cZi-DBS compared with STN-DBS for two groups with eight patients in each. While the magnitudes of the differences for group means were small, the responses at individual patient level were remarkably consistent. The subtle differences found for mean intensity suggest that STN-DBS and cZi-DBS may influence voice intensity differently. A related study that used essentially the same patient groups has also shown a differential response between cZi- and STN-DBS for articulatory proficiency [31]. It will be important to confirm these differential effects and their consistency at individual level in studies with larger patient numbers.

## Figures and Tables

**Table 1:** Characteristics of patients in the two surgical groups. There were no statistical differences between the groups for age, duration since diagnosis, or any of the UPDRS scores.

Patient	Uni/bilateral	Gender	Age at operation (y)	Duration since diagnosis (y)	UPDRS III		Speech UPDRS III Item 18	
					Off med	On med	Off med	On med
STN1	bi	M	68	9	52	24	2	2
STN2	uni	M	65	7	33	16	1	0
STN3	bi	M	58	7	39	18	2	1
STN4	bi	M	64	5	40	16	0	0
STN5	bi	F	51	8	32	6	1	0
STN6	bi	F	53	8	44	21	1	1
STN7	bi	M	70	4	57	36	2	1
STN8	uni	M	72	6	35	22	0	0

Median (range)			65 (51–72)	7 (4–9)	39.5 (32–57)	19.5 (6–36)	1.0 (0–2)	0.5 (0–2)

czI1	bi	F	66	10	29	15	1	1
czI2	bi	M	70	2	40	42	1	1
czI3	bi	M	71	7	43	24	2	1
czI4	bi	M	62	5	50	27	2	1
czI5	bi	M	49	4	30	14	1	1
czI6	bi	M	50	6	31	10	1	0
czI7	bi	F	62	5	31	16	0	0
czI8	bi	M	52	10	58	25	2	1

Median (range)			62.5 (49–71)	5.5 (2–10)	35.5 (29–58)	20.0 (10–42)	1.0 (0–2)	1.0 (0-1)

## Appendix

Reading passage

En pojke kom en dag inspringande på en bondgård och undrade om han kunde få låna en spade. Bonden frågade vad han skulle ha spaden till. Pojken svarade att hans bror hade ramlat i ett träsk och han måste gräva upp honom. “Hur djupt har han ramlat i?” frågade bonden. “Upp till vristerna”, blev svaret. “Men då han kan väl gå därifrån utan din hjälp så då behöver du väl ingen spade?” Pojken såg förtvivlad ut och sa: “Jo, men ni förstår, han ramlade i med huvudet före.” (Ett svårt fall)

## References

[1] Murdoch BE (2010). Surgical approaches to treatment of Parkinson’s disease: implications for speech function. *International Journal of Speech-Language Pathology*.

[2] Deuschl G, Herzog J, Kleiner-Fisman G (2006). Deep brain stimulation: postoperative issues. *Movement Disorders*.

[3] Plaha P, Ben-Shlomo Y, Patel NK, Gill SS (2006). Stimulation of the caudal zona incerta is superior to stimulation of the subthalamic nucleus in improving contralateral parkinsonism. *Brain*.

[4] Blomstedt P, Fytagoridis A, Tisch S (2009). Deep brain stimulation of the posterior subthalamic area in the treatment of tremor. *Acta Neurochirurgica*.

[5] Hartelius L, Svensson P (2004). Speech and swallowing symptoms associated with Parkinson’s disease and multiple sclerosis: a survey. *Folia Phoniatrica et Logopaedica*.

[6] Logemann JA, Fisher HB, Boshes B, Blonsky ER (1978). Frequency and cooccurrence of vocal tract dysfunctions in the speech of a large sample of Parkinson patients. *Journal of Speech and Hearing Disorders*.

[7] Sapir S, Pawlas AA, Ramig LO (2001). Voice and speech abnormalities in Parkinson disease: relation to severity of motor impairment, duration of disease, medication, depression, gender, and age. *Journal of Medical Speech-Language Pathology*.

[8] Fox CM, Ramig LO (1997). Vocal sound pressure level and self-perception of speech and voice in men and women with idiopathic Parkinson disease. *American Journal of Speech-Language Pathology*.

[9] Ho AK, Iansek R, Marigliani C, Bradshaw JL, Gates S (1998). Speech impairment in a large sample of patients with Parkinson’s disease. *Behavioural Neurology*.

[10] Schulz GM, Grant MK (2000). Effects of speech therapy and pharmacologic and surgical treatments on voice and speech in Parkinson’s disease: a review of the literature. *Journal of Communication Disorders*.

[11] Ho AK, Iansek R, Bradshaw JL (2001). Motor instability in Parkinsonian speech intensity. *Neuropsychiatry, Neuropsychology and Behavioral Neurology*.

[12] Rosen KM, Kent RD, Duffy JR (2005). Task-based profile of vocal intensity decline in parkinson’s disease. *Folia Phoniatrica et Logopaedica*.

[13] Hammer MJ, Barlow SM, Lyons KE, Pahwa R (2010). Subthalamic nucleus deep brain stimulation changes speech respiratory and laryngeal control in Parkinson’s disease. *Journal of Neurology*.

[14] Suchowersky O, Gronseth G, Perlmutter J, Reich S, Zesiewicz T, Weiner WJ (2006). Practice parameter: neuroprotective strategies and alternative therapies for Parkinson disease (an evidence-based review): Report of the Quality Standards Subcommittee of the American Academy of Neurology. *Neurology*.

[15] D’Alatri L, Paludetti G, Contarino MF, Galla S, Marchese MR, Bentivoglio AR (2008). Effects of bilateral subthalamic nucleus stimulation and medication on Parkinsonian speech impairment. *Journal of Voice*.

[16] Dromey C, Kumar R, Lang AE, Lozano AM (2000). An investigation of the effects of subthalamic nucleus stimulation on acoustic measures of voice. *Movement Disorders*.

[17] Gentil M, Chauvin P, Pinto S, Pollak P, Benabid AL (2001). Effect of bilateral stimulation of the subthalamic nucleus on parkinsonian voice. *Brain and Language*.

[18] Gentil M, Pinto S, Pollak P, Benabid AL (2003). Effect of bilateral stimulation of the subthalamic nucleus on parkinsonian dysarthria. *Brain and Language*.

[19] Klostermann F, Ehlen F, Vesper J (2008). Effects of subthalamic deep brain stimulation on dysarthrophonia in Parkinson’s disease. *Journal of Neurology, Neurosurgery and Psychiatry*.

[20] Pinto S, Gentil M, Krack P (2005). Changes induced by Levodopa and subthalamic nucleus stimulation on Parkinsonian speech. *Movement Disorders*.

[21] Pützer M, Barry WJ, Moringlane JR (2008). Effect of bilateral stimulation of the subthalamic nucleus on different speech subsystems in patients with Parkinson’s disease. *Clinical Linguistics and Phonetics*.

[22] Rousseaux M, Krystkowiak P, Kozlowski O, Özsancak C, Blond S, Destée A (2004). Effects of subthalamic nucleus stimulation on parkinsonian dysarthria and speech intelligibility. *Journal of Neurology*.

[23] Santens P, De Letter M, Van Borsel J, De Reuck J, Caemaert J (2003). Lateralized effects of subthalamic nucleus stimulation on different aspects of speech in Parkinson’s disease. *Brain and Language*.

[24] Tripoliti E, Limousin P, Tisch S, Bottell E, Hariz M (2006). Speech in Parkinson’s disease following subthalamic nucleus deep brain stimulation: preliminary results. *Journal of Medical Speech-Language Pathology*.

[25] Tripoliti E, Zrinzo L, Martinez-Torres I (2011). Effects of subthalamic stimulation on speech of consecutive patients with Parkinson’s disease. *Neurology*.

[26] Wang E, Verhagen Metman L, Bakay R, Arzbaecher J, Bernard B (2004). Effect of unilateral electrostimulation of the subthalamic nucleus on speech in Parkinson’s disease. *Movement Disorders*.

[27] Wang E, Verhagen Metman L, Bakay R, Arzbaecher J, Bernard B (2003). The effect of unilateral electrostimulation of the subthalamic nucleus on respiratory/phonatory subsystems of speech production in Parkinson’s disease—a preliminary report. *Clinical Linguistics and Phonetics*.

[28] Wang E, Verhagen Metman L, Bakay R, Arzbaecher J, Bernard B, Corcos DM (2006). Hemisphere-specific effects of subthalamic nucleus deep brain stimulation on speaking rate and articulatory accuracy of syllable repetitions in Parkinson’s disease. *Journal of Medical Speech-Language Pathology*.

[29] Van Lancker Sidtis D, Rogers T, Godier V, Tagliati M, Sidtis JJ (2010). Voice and fluency changes as a function of speech task and deep brain stimulation. *Journal of Speech, Language, and Hearing Research*.

[30] van Doorn J, Schalling E, Hartelius L, Asplund A (2010). Stimulation of zona incerta in Parkinson’s disease: a first look at speech outcomes. *Movement Disorders*.

[31] Karlsson F, Unger E, Wahlgren S Deep brain stimulation of caudal zona incerta and subthalamic nucleus in patients with Parkinson’s disease: effects on diadochokinetic rate.

[32] Blomstedt P, Hariz MI (2006). Are complications less common in deep brain stimulation than in ablative procedures for movement disorders?. *Stereotactic and Functional Neurosurgery*.

[33] Asplund A, Murdoch B, Goozee J, Whelan B-M, Docking K How loud was it? A calibration system for voice recording in clinical and research applications.

[34] Boersma P, Weening D Praat: doing phonetics by computer (Version 5.1.32). http://www.praat.org/.

[35] Tjaden K, Watling E (2003). Characteristics of diadochokinesis in multiple sclerosis and Parkinson’s disease. *Folia Phoniatrica et Logopaedica*.

[36] De Letter M, Van Borsel J, Boon P, De Bodt M, Dhooge I, Santens P (2010). Sequential changes in motor speech across a levodopa cycle in advanced Parkinson’s disease. *International Journal of Speech-Language Pathology*.

[37] Goberman A, Coelho C, Robb M (2002). Phonatory characteristics of Parkinsonian speech before and after morning medication: the ON and OFF states. *Journal of Communication Disorders*.

[38] Jiang J, Lin E, Wang J, Hanson DG (1999). Glottographic measures before and after levodopa treatment in Parkinson’s disease. *The Laryngoscope*.

